# Hemisection of Grossly-Decayed Teeth With Socket Preservation: A Case Report

**DOI:** 10.7759/cureus.96574

**Published:** 2025-11-11

**Authors:** Deepika Deepika, Mutiur Rahman, Mohan Rawat, Ajay Nagpal, Shivendra Choudhary

**Affiliations:** 1 Department of Conservative Dentistry and Endodontics, All India Institute of Medical Sciences Patna, Patna, IND; 2 Department of Conservative Dentistry and Endodontics, Kanti Devi Dental College &amp; Hospital, Mathura, IND; 3 Department of Periodontology and Implantology, Kanti Devi Dental College &amp; Hospital, Mathura, IND; 4 Dentistry, All India Institute of Medical Sciences Patna, Patna, IND

**Keywords:** bone graft, crown lengthening, decayed teeth, extraction socket preservation, hemisection, socket preservation, subgingival caries

## Abstract

Hemisection of a molar is a surgical procedure involving the removal or separation of one root together with its corresponding portion of the crown. This approach allows for the preservation of the remaining tooth structure and surrounding alveolar bone, while providing support for subsequent fixed prosthetic rehabilitation. The present case report describes the successful application of hemisection to retain a severely compromised mandibular first molar with periodontal and periapical involvement. Following prosthetic rehabilitation, the treatment achieved a favourable outcome. Through meticulous case selection, careful treatment planning, and precise surgical execution, the adverse consequences associated with tooth loss were effectively avoided.

## Introduction

Tooth loss can occur due to various factors such as dental caries, periodontal disease, trauma, infection, malignancies, or failed endodontic treatments, and it can have negative consequences on both the remaining teeth and the overall well-being of patients [1]. The field of dentistry has seen advancements that focus on tooth preservation through a multidisciplinary approach. One such treatment option is hemisection, which combines principles from prosthodontics, oral surgery, endodontics, periodontics, and restorative dentistry [2]. Hemisection is a conservative method of preserving a tooth. It involves sectioning or amputating a root, also referred to as root sectioning or bisection. This approach allows for the preservation of the tooth structure and alveolar bone and is cost-saving compared to other treatment alternatives [3].

Indications for hemisection include severe bone loss affecting one or more roots or the furcation area where other periodontal surgical options are not feasible. Additional indications include closely approximated roots that limit periodontal access and irreparable root damage resulting from fractures, perforations, resorption, calcified canals, or retained fractured instruments. Conversely, hemisection is contraindicated in cases with inadequate bone support for the remaining root, inability to perform successful endodontic treatment, root fusion preventing separation, unfavourable restorative or prosthetic design, or poor oral hygiene and patient compliance [4].

In the present case, hemisection was performed due to significant destruction of the distal root with subgingival caries and Glickman grade III furcation defect. The treatment involved extracting the affected distal root and crown with socket preservation and performing crown lengthening on the mesial root. The remaining tooth structure was rehabilitated with a metal post and crown and was utilised as an abutment for a crown-and-bridge prosthesis, following adjustment of the occlusal contacts to a favourable position. This case report demonstrates a combined endodontic-periodontal-prosthetic management of a mandibular molar with furcation involvement using socket preservation and long-term functional rehabilitation.

## Case presentation

**Figure 1:**
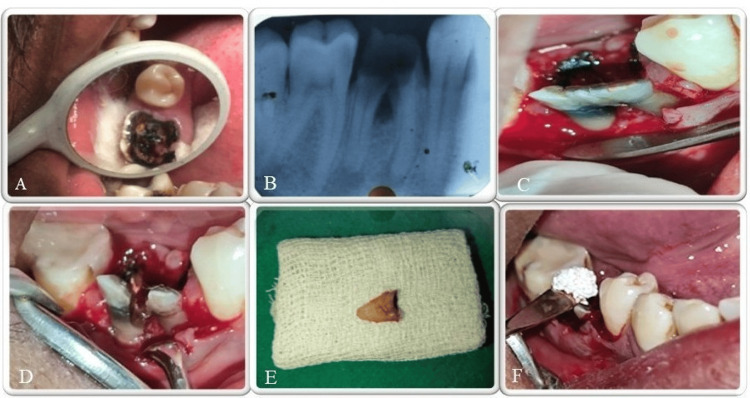
Pre- and intraoperative images A) Pre-operative clinical picture; B) Pre-operative radiograph #46; C) Envelope flap raised; D) Sectioning of #46; E) Extracted distal root; F) Bone graft placed in the distal root socket.

A 42-year-old male patient reported to the Department of Conservative Dentistry and Endodontics with a chief complaint of pain and decay in the lower right back tooth region, persisting for the past month. The patient experienced mild, intermittent pain that was aggravated by mastication. Intraoral clinical examination revealed a grossly carious lesion extending subgingivally in tooth #46 with a pocket probing depth of 8 mm in the furcation area (Figure 1A). 

However, no mobility was observed in #46. Mesio-proximal and occlusal caries were present in #47. Both #46 and #47 were tender on vertical percussion. Electric pulp testing gave a delayed response for both #46 and #47. Radiographic findings revealed radiolucency involving the enamel, dentin, and pulp in #46 along with grade III furcation involvement (Figure 1B). 

Periapical radiolucency involving both mesial and distal roots was present with respect to #46. Widening of the periodontal space was seen with respect to #47. Based on the history, clinical and radiographic examination, a diagnosis of pulp necrosis with chronic apical abscess associated with an endodontic-periodontal involvement in relation to tooth #46 was made, along with symptomatic irreversible pulpitis with apical periodontitis for #47. The patient was counselled regarding the condition, questionable prognosis of teeth, and feasible treatment options, including extraction with subsequent implant placement. After considering the alternatives, the patient opted for hemisection as the preferred treatment modality. Informed consent was obtained from him for the proposed treatment plan. After determining the working length of the canals in tooth #46, the canals were irrigated with 3% sodium hypochlorite (Parcan, Septodont, France) and 0.9% normal saline for removal of the pulp tissue and debris. Root canal shaping was completed with the Hyflex CM 25/.04 (Coltene-Whaledent, Allstetten, Switzerland) rotary file system, and an inter-appointment calcium hydroxide dressing (Prime RC Cal, Prime Dental Products Pvt. Ltd., Thane, India) was placed for 21 days. The mesio-buccal and mesio-lingual root canals of #46 were obturated with gutta percha.

**Figure 2:**
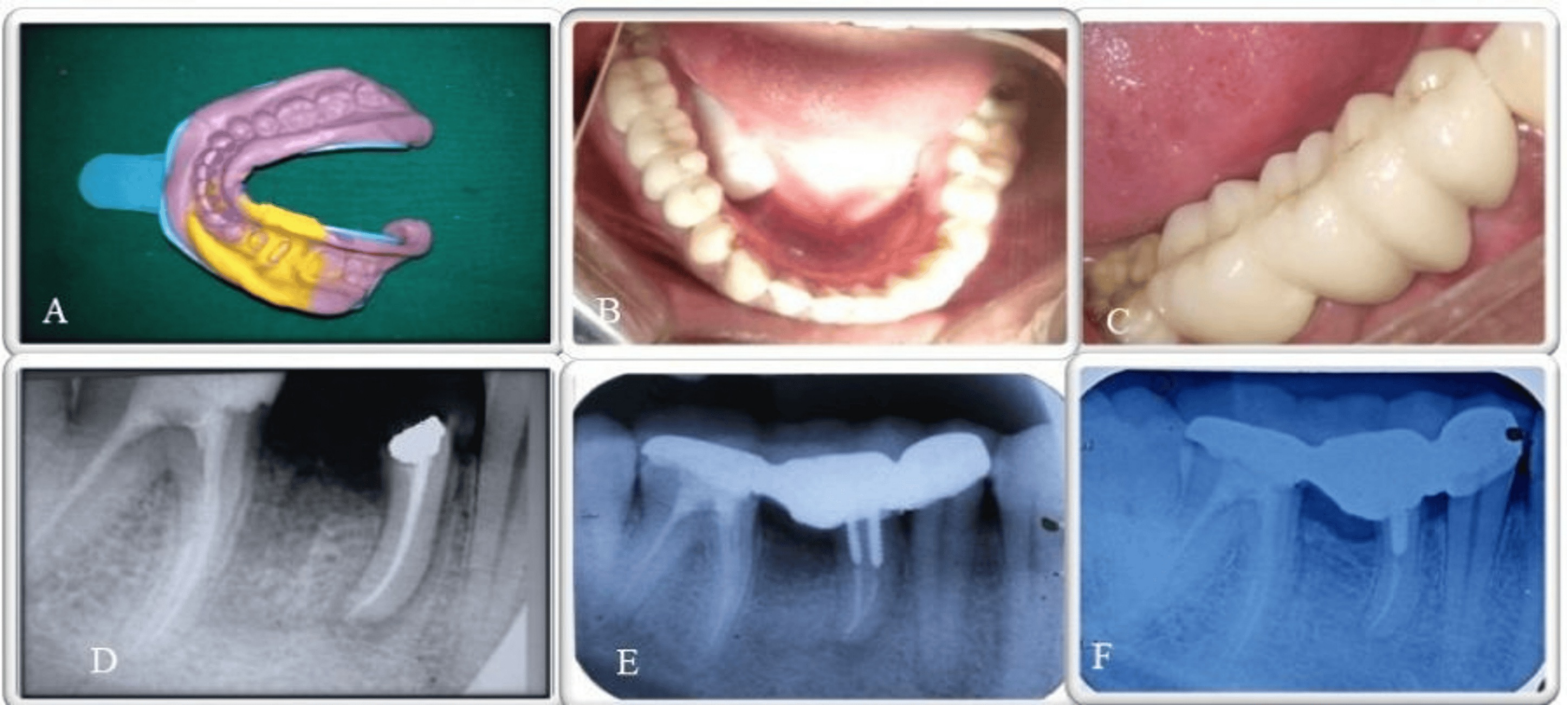
Post-operative images A) Polyvinyl siloxane impression of lower arch; B) Post-operative occlusal view of lower arch; C) Post-operative lateral view; D) One month follow-up radiograph; E) One year follow-up radiograph; F) Three-year follow-up radiograph.

In the next visit, a full-thickness flap was reflected following a crevicular incision that extended from the second premolar to the second molar, all performed under local anaesthesia (Figure 1C). Using a long-shank tapered fissure carbide bur, a vertical cut was made down toward the bifurcation area (Figure 1D). A fine probe confirmed complete separation of the tooth structure. The distal root of #46 was then extracted (Figure 1E), and the socket was curetted and thoroughly irrigated with saline to remove any bony chips. For crown lengthening, a 2-3 mm osseous reduction was made. Osseous surgery was done to obtain a positive architecture. Sterile synthetic hydroxyapatite and β-tricalcium phosphate bone graft (Sybograf^TM^ Plus, Eucare Pharmaceuticals Private Limited, Chennai, India) was placed in the socket of distal root for socket preservation (Figure 1F). The flap was repositioned using interrupted sutures (Figure 1F) and periodontal pack (Coe-Pak^TM^ GC America Inc., Alsip, IL, USA) was placed. The occlusal table was minimized to ensure masticatory forces were directed along the long axis of the retained mesial root. At the one-month follow-up visit, healing was uneventful, with no signs of mobility or persistent pain, and a noticeable reduction in probing depth was observed. Metal post and core, size short no-2 (Dental Concepts, Nagpur, India), were placed in the mesio-buccal and mesio-lingual root canals of #46. Tooth preparation was performed on #45, #46 and #47, followed by the making of a putty-wash impression of the mandibular arch using putty and light-body addition silicon impression material (GC Flexceed Putty and Light Body Kit, GC India Dental Pvt. Ltd., Telangana, India) (Figure 2A).

A fixed partial denture (FPD) incorporating a ridge-lap pontic design was fabricated to restore teeth #45, #46, and #47. The prosthesis was luted using Type I Glass Ionomer Cement (AquaCem, Dentsply, Charlotte, USA) (Figures 2B, 2C). Subsequent follow-ups were conducted at one month, one year, and three years (Figures 2D-2F). Radiographic evaluation at the three-year follow-up demonstrated the absence of periodontal ligament widening and substantial bone fill within the defect, reflecting favourable periodontal healing.

## Discussion

The endodontic approach of hemisection involves the partial removal of a multirooted tooth while also treating the remaining roots with root canal therapy and restoring them using appropriate restorative materials. In the present case, the distal root was extracted, and the mesial root was retained. In comparison to the mesial root in mandibular molars, there is limited literature available on distal root resection due to its anatomical complexity [5,6]. The success of hemisected molars in the long term relies on various interconnected factors. These include the periodontal health of the tooth, the anatomy of the root, the quality of oral hygiene maintenance, the effectiveness of endodontic and restorative treatments, and the surgical procedure [7]. In this case, socket preservation was performed on the extracted distal root. The benefits of socket preservation therapy are primarily associated with improved restorative and aesthetic results, as well as the effective maintenance of healthy soft tissues [8]. As per the findings of Park et al. [9], hemisection is a reliable treatment option for molars with uncertain prognosis. It has the potential to maintain the teeth with minimal bone loss for an extended period, provided the patient maintains excellent dental hygiene. Basten et al. [10] conducted a study reporting that 92% of resected molars survived over an average period of 12 years. Failures were primarily attributed to recurrent caries or endodontic and strategic reasons. However, Erpenstein presented less favourable results for hemisected molars, with an overall failure rate of 20.6%, primarily attributed to pathologic apical factors [11]. Lin et al. reported successful hemisection of a mandibular molar with distal root involvement, where preservation of the mesial root and prosthetic rehabilitation using a zirconia fixed partial denture yielded favourable functional outcomes [12].

In our case, after three years of follow-up, the patient's condition improved significantly with improved oral hygiene and the absence of further bone loss or tooth mobility. As a result, the preferred treatment approach was to perform a hemisection with maintenance of the roots. This procedure was chosen to preserve the functional dentition and ensure long-term oral health.

## Conclusions

Hemisection marks a paradigm shift toward the preservation of natural tooth structure and supporting alveolar bone, which are critical for maintaining periodontal health and preventing collapse of the dental arch. Beyond its biological and functional advantages, it also provides practical benefits, offering substantial cost savings and alleviating the psychological distress often associated with complete tooth loss or implant replacement. When appropriate selection criteria and meticulous endodontic, periodontal, surgical, and restorative protocols are followed, it stands out as a conservative and reliable treatment modality. It allows for the preservation of natural dentition, minimises financial burden, reduces psychological impact, and maintains occlusal harmony in the management of severely compromised multirooted teeth.

This case report presents successful long-term retention through an integrated endodontic, periodontal, and prosthetic management. Future need for larger longitudinal studies to validate combined hemisection-socket preservation protocols.
